# Endocannabinoid-Epigenetic Cross-Talk: A Bridge toward Stress Coping

**DOI:** 10.3390/ijms21176252

**Published:** 2020-08-29

**Authors:** Francesco Rusconi, Tiziana Rubino, Elena Battaglioli

**Affiliations:** 1 Department of Medical Biotechnology and Translational Medicine, Università degli Studi di Milano—Via Fratelli Cervi 93, 20090 Segrate (MI), Italy; elena.battaglioli@unimi.it; 2 Department of Biotechnology and Life Sciences, Università degli Studi dell’Insubria—Busto Arsizio (VA), 21052 Busto Arsizio, Italy; tiziana.rubino@uninsubria.it

**Keywords:** endocannabinoid system, epigenetics, homeostasis, psychiatric disorders, 2-arachidonoylglycerol (2-AG), Lysine Specific Demethylase 1, Hypothalamus-Pituitary-Adrenocortical (HPA)

## Abstract

There is no argument with regard to the physical and psychological stress-related nature of neuropsychiatric disorders. Yet, the mechanisms that facilitate disease onset starting from molecular stress responses are elusive. Environmental stress challenges individuals’ equilibrium, enhancing homeostatic request in the attempt to steer down arousal-instrumental molecular pathways that underlie hypervigilance and anxiety. A relevant homeostatic pathway is the endocannabinoid system (ECS). In this review, we summarize recent discoveries unambiguously listing ECS as a stress coping mechanism. As stress evokes huge excitatory responses in emotional-relevant limbic areas, the ECS limits glutamate release via 2-arachydonilglycerol (2-AG) stress-induced synthesis and retrograde cannabinoid 1 (CB1)-receptor activation at the synapse. However, ECS shows intrinsic vulnerability as 2-AG overstimulation by chronic stress rapidly leads to CB1-receptor desensitization. In this review, we emphasize the protective role of 2-AG in stress-response termination and stress resiliency. Interestingly, we discuss ECS regulation with a further nuclear homeostatic system whose nature is exquisitely epigenetic, orchestrated by Lysine Specific Demethylase 1. We here emphasize a remarkable example of stress-coping network where *transcriptional* homeostasis subserves synaptic and behavioral adaptation, aiming at reducing psychiatric effects of traumatic experiences.

## 1. Introduction

When the sensation of feeling under threat elicits an exceptional psychological arousal, an individual gets involved in a fearful domain of emotions and autonomic responses that cumulatively goes under the definition of stress response. This is evolutionarily due to the tendency—because the aim is saving individuals’ life—to field all the available metabolic resources, a protective mechanism that does not come without a price. In the central nervous system, threat is perceived by senses and transduced via excitatory glutamate signaling, which is also instrumental to engrave a memory trace of the negative experience. When glutamate release overpasses a given quantitative threshold, it triggers a specific stress response promoting the activation of the Hypothalamic-Pituitary-Adrenocortical (HPA) axis and the autonomic nervous system (ANS). This threshold can be overpassed (i) when many sensory inputs of diverse nature converge on integrative brain centers, (ii) when we are hit by a single stimulus (auditory, visive, tactile or olfactive) of exceptionally high intensity or (iii) when an even very mild stimulus is endowed with a strong negative emotional component in terms of associative recall of reinforcing circuitries. On the contrary, milder glutamate release in response to non-threatening environmental stimuli induces normal reactions to reward-related, inoffensive, or anodyne events. In the first case, excitatory circuitries of brain areas involved in stress response, amygdala, hippocampus and prefrontal cortex among others, undergo a demanding, potentially dangerous engagement in terms of glutamate load and consequent neuronal activation. In this case, glutamate responses might exceed the protective scope (for instance a balanced fight or flight reaction) or, in a worse scenario, they might lead to excitotoxicity-related circuitry disruption [1,2]. This is why such responses evolved along with specific, circuitry protective, homeostatic mechanisms among which the best characterized is the endocannabinoid system (ECS) [3]. The ECS is widely distributed in the central nervous system (CNS), constituting a complex signaling system that subserves multiple modes of synaptic transmission modulation. It is expressed at some synapses in all brain regions that are important for the processing of anxiety, fear and stress [4]. In cortical areas (including the cerebral cortex, hippocampus and cortical parts of the amygdala), CB1 receptor is expressed at higher level in cholecystokinin (CCK)-positive GABAergic interneurons, and at lower level in glutamatergic neurons. However, CB1 modulation in glutamatergic neurons has been shown to play an important role in the control of synaptic transmission and neuronal excitability [5,6]. The ECS represents, therefore, a negative synaptic feedback system activated by different neurotransmitters including glutamate, that acts to constrain neurotransmitter activity within stable and adaptive physiological ranges [4]. Since glutamate responses are not limited to synapse but continue into the nucleus where they promote cellular morphological remodeling, hence memory formation, another set of homeostatic mechanisms are devoted to buffer activity-dependent transcription through the rapid modulation of chromatin structure. This is mediated by transient modification of the level or activity of specific epigenetic modifiers [7,8,9]. 

Notwithstanding homeostatic mechanisms, environmental stress is always potentially toxic, as well as a prominent risk factor for psychiatric disorders [10]. Indeed, even if the majority of individuals can cope with stressful events neutralizing their harmful consequences, a substantial proportion of the human population cannot, being defined as vulnerable and developing long lasting signs of mental illness in response to trauma. In this review, we summarize the main synaptic, endocannabinoid-related, and epigenetic mechanisms aimed at protecting neurons and circuitry from glutamate stress-induced overload. We will also give a perspective on how these topologically distinct (synaptic-nuclear) homeostatic domains could regulate each other. To conclude we propose a novel standpoint on how allostatic overload of an epigenetic protecting mechanism—in response to chronic stress—possibly concurs to loss of homeostatic function. 

## 2. 2-AG Modulation of Stress Adaptation in the Hippocampus Is Mainly Devoted to Excitatory Control

Neurons are continuously involved in fine-tuning their input/output responses. The ECS represents a prominent synaptic mechanism absolving to this task, restraining when needed GABA release to increase glutamatergic transmission, but also limiting glutamate release when input is too high. ECS homeostatic function is specifically involved in stress response [11], a demanding domain of neuronal adaptation. 

The ECS is a lipid-mediated signalling system whose existence has been discovered in the last decade of the last century [12]. It is composed by lipid ligands (the most studied are 2-arachidonoylglycerol (2-AG) and anandamide, in short AEA), by two G protein-coupled receptors, cannabinoid receptors CB1 and CB2, and enzymes involved in the biosynthesis and degradation of the ligands. Although initial evidence suggested AEA as the prominent player in the regulation of stress response and anxiety behaviour (see Morena et al., 2016 for review [11]), increasing evidence supports a role for 2-AG in stress adaptation and in controlling anxiety [12]. Thus, 2-AG is a full agonist at CB1 and CB2 receptors and functions as an endocannabinoid retrograde signaling molecule. Indeed, postsynaptically generated 2-AG mediates retrograde signaling mechanisms aimed at inhibiting stress-evoked neurotransmitter release, called depolarization-induced suppression of excitation (DSE, suppression of glutamate release) and inhibition (DSI, suppression of GABA release) at excitatory and inhibitory synapses, respectively [13,14]. In actuality, 2-AG is synthesized by diacylglycerol lipase (DAGL), and it is degraded by monoacylglycerol lipase (MAGL), located presynaptically, but can also be hydrolysed by alpha-beta hydrolase domain-containing protein 6 (ABHD6), that is instead located postsynaptically. Accordingly, DSE and DSI are abolished in DAGLα knockout mice [15,16] and prolonged in MAGL-deficient mice [17,18].

Accumulating evidence indicates that the endocannabinoid signalling in the brain plays a central role in the control of stress, fear and anxiety [4]. The exposure to aversive stimuli or stress initially elicits glutamate release in the hippocampus [19], as well as a delayed endocannabinoid-mediated homeostatic signalling [20]. Remarkably, this stress-mediated enhancement of excitatory activity can be counteracted by the elevation of 2-AG levels [20,21]. This event has an important function in reducing the expression of anxiety-like behaviours. Indeed, genetic and pharmacological inhibition of 2-AG synthesis increases anxiety-like behaviour in rodent models, while pharmacological inhibition of 2-AG degradation, and thus increased 2-AG levels, shows anxiolytic effects [12].

Restraint stress has been shown to decrease AEA and increase 2-AG immediately after stress exposure in the hippocampus [20]. AEA returns to control level within 30 minutes, whereas 2-AG remains elevated for a longer time. Acute restraint stress also enhances DSI in the hippocampus of male rats [20]. This effect takes time to develop, being evident only 30 min after the end of the restraint period and is blocked by glucocorticoid receptor antagonists. The authors suggested that DSI is mediated by 2-AG and that stress-induced mobilization of 2-AG signalling could represent an adaptive response to acute stress, which would help maintain emotional and behavioural flexibility in the face of aversive stimuli [20]. However, since 2-AG is involved in DSE too, an interesting question arises about whether DSI is the only short-term plasticity mechanism induced by stress that rely on 2-AG signalling, which seems not to be the case. Indeed, it is possible that also DSE could contribute to 2-AG modulation of stress response. To induce DSE, CB1 receptors are needed on glutamatergic neurons [22]. Remarkably, CB1 receptors on glutamatergic neurons are also needed for the fear-alleviating effect of endocannabinoids [21,23], thus suggesting that unrestrained glutamate release may account for the sustained fear responses observed in CB1-deficient mice [24,25]. Along this line, CB1 receptor activation on glutamatergic neurons is required for the anxiolytic effect of low doses of cannabinoids [26]. Furthermore, to investigate the functions of 2-AG in hippocampal glutamatergic neurons *in vivo*, MAGL was selectively overexpressed in these neurons [5]. Such a manipulation attenuated 2-AG-mediated DSE without significantly affecting 2-AG action on GABAergic transmission in CA1 pyramidal neurons and induced an anxiety-like behaviour in animals. All these data point toward hippocampal glutamatergic 2-AG signaling as an essential component of adaptation to aversive situations.

## 3. Epigenetic Homeostatic System Protects against Excessive Excitatory Effects of Stress

As anticipated, environmental stress elicits glutamatergic activation within brain areas involved in cognitive and emotional processing [27]. Besides immediate synaptic modifications, independent of transcriptional and translational processes and instrumental to trigger immediate stress response reactions, nuclear pathways are induced to consolidate an operative, cognitive and affective memory engram of the negative experience under the form of long-lasting changes of neuronal physiology. Transcriptional responses to a stressor—resulting from glutamate signal transduction—always implicates as first step, Immediate Early Genes (IEGs) activation in the hippocampus, amygdala and prefrontal cortex, promoting environmental adaptation by means of modified reactions to further stimuli [7]. As said, glutamatergic circuitries are *per se* vulnerable to overstimulation, but also their transduction, involving cognitive and emotional-relevant behavioral read-outs must be kept under tight control [7]. This is why also on a transcriptional point of view, initial stress responses must be constrained within physiological ranges, dosing intensity and duration of their elicited transcriptional waives, starting from the IEGs [28,29]. The study of nuclear processes devoted to buffering experience-evoked transcription represents an emerging field of neurobiology research. Homeostatic transcriptional plasticity (HTP) *bridle* neuroplastic and memory-instrumental transcription, flanking and integrating those processes underlying homeostatic synaptic plasticity (HSP), in the remarkable task to constrain brain activity into stable physiological and adaptive ranges [30]. Notably, and consistently with its functional definition as an interface between environment and gene expression, the neuronal epigenome is the substrate of mentioned transcriptional homeostasis. Only a few examples of such processes have been suggested so far [7], all related to transient, stress-induced, negative epigenetic regulation in the hippocampus within a paradigm-specific window of stress response that follows the traumatic event. These include increase of H3K9 methyltransferase Suv39H2 [31], DNA methyl transferase 3a (Dnmt3a) [28], and Lysine Specific Demethylase 1 (LSD1) [32], all involved in negative transcriptional regulation. Interestingly, for what concerns Dnmt3a and LSD1, preferential targets were indeed the IEGs. Consistently and within the same window of acute stress response, also negative epigenetic marks are homeostatically increased in the hippocampus. Strikingly, EGR1 promoter methylation increases in response to foot shock paradigm as early as 30 minutes after the trauma, possibly buffering EGR1 transactivation upon stress [33]. Moreover, an independent work [28] recently showed how, in conditions of high substrate availability through supplementation of *S*-adenosyl methionine (SAM, the methyl donor, substrate of DNA methyltransferases), the same promoter region of EGR1 (as well as c-FOS promoter) undergoes Dnmt3a-mediated DNA methylation. Interestingly, DNA methylation prevents forced swim stress-induced transactivation of EGR1 and c-FOS [28]. With these experiments, the authors showed that limiting IEGs transactivation concurs to define an *adaptive* onset of stress-related behavioral reactions [28], unravelling the importance of homeostatic transcriptional mechanisms to healthy behavioral responses.

Another pathway of transcriptional homeostasis includes a neurospecific alternative splicing-based mechanism regulating the activity of Lysine Specific Demethylase 1 (LSD1). LSD1 is a highly conserved (from yeast to humans), ubiquitously expressed transcriptional corepressor, removing epigenetic marks of active transcription, namely H3K4me1/2 [34,35]. Interestingly, a brain-restricted *LSD1* splicing isoform named neuroLSD1 has evolved in mammals to tune-down LSD1 activity in neurons [7,32,36], through a microexon-based mechanism [37]. Microexons have been proposed to exert a switch-like modification of protein function mainly modulating protein-protein interaction instrumentally to transient changes in neuronal interactome [38,39,40]. NeuroLSD1 includes an additional 12 nucleotide-long microexon, the E8a, encoding an additional stretch of amino acids (DTVK) that dramatically impacts LSD1 function [36]. Indeed, neuroLSD1 is in vivo devoid of catalytic activity and it cannot bind its core cofactors CoREST and HDAC2 [41], representing therefore a dominant negative isoform unable to repress transcription. Interestingly, in the brain, LSD1 and neuroLSD1 co-regulate activity-evoked gene transcription, concurring to memory consolidation and regulating anxiety-like profile [32,42]. Relevantly, among the best characterized LSD1 and neuroLSD1 targets, the Immediate Early Genes (IEGs) again represent a forefront category. Taking into consideration the opposing coregulatory role of LSD1 and neuroLSD1, their relative amount in neurons impacts IEGs responsivity in the hippocampus [32,43]. Indeed, to be transactivated in response to stimuli, the IEGs require a transcription-permissive LSD1/neuroLSD1 ratio in which neuroLSD1 counteracts the repressive H3K4 demethylase activity of LSD1 over their common IEGs targets including EGR1, NPAS4, NR4A1 and c-FOS [32,42]. In particular, it was shown that a single session of social defeat stress exerts a transient, hours-long splicing-mediated decrease of neuroLSD1 generating a transcription “non-permissive” LSD1/neuroLSD1 ratio aimed at transiently restraining IEGs transcription. Homeostatic relevance of such a mechanism can be inferred considering low anxiety-like profile of neuroLSD1 haplo-insufficient mice, a model of isoform-specific knock down fully preserving LSD1 expression [32]. When administered with the psychosocial stress, these mice do not efficiently induce the neuroplastic program of gene expression, as seen in terms of c-FOS and EGR1 transactivation in the hippocampus. These experiments suggested that, similarly to c-FOS and EGR1 stress-induced DNA methylation [28], also physiologically increased LSD1 activity (by decreased neuroLSD1 levels) might be aimed at limiting behavioral stress responses [32].

Collectively, these data indicate that transcriptional homeostatic mechanisms exist in the nucleus of hippocampal neurons, being triggered in response to different stressors, in different species (mouse and rat), limiting the same typology of neuroplastic gene transcription with converging epigenetic strategies, all finalized to decreasing negative behavioral stress-induced short- and long-term alterations. As these mechanisms concurring to *epigenetic homeostatic system* are commonly promoted by stress-induced glutamatergic neuron depolarization in the hippocampus, we propose to collectively refer to them as depolarization-induced suppression of transcription (DST).

## 4. ECS and Epigenetic Homeostatic System Cross-Regulation

So far, we have described two homeostatic systems pertaining to distinct cellular domains of glutamatergic neurons: the long-known widely-studied endocannabinoid system, whose main importance in stress-response termination may rely on the process of depolarization-induced suppression of excitation (DSE) [5,6] and the epigenetic homeostatic system, involved in depolarization-induced suppression of transcription (DST) [7]. Although seemingly independent, these systems are similarly involved in limiting short- and long-term behavioral effects of stress via decreasing inherent neuroplasticity and learning-instrumental transcription. In other words, they tend to tune down glutamate transduction (primed by glutamate receptor activation at the synapse) uncoupling environmental stimuli from experience-evoked transcription. DST is aimed at limiting morphostructural memory-encoding changes of excitatory neurons, ultimately protecting from excessive strengthening of anxiety-like response and keeping arousal at adaptive levels [7].

We recently documented that functional cooperativity between ECS and epigenetic homeostatic system is not limited to an outcome convergence toward limiting synaptic and neuroplastic effects of stress. In particular, a notable feedforward transcriptional mechanism orchestrated by LSD1 is aimed at reinforcing endocannabinoid-mediated suppression of stress-induced glutamate release in the hippocampus. As previously described, endocannabinoid 2-AG is synthesized in dendritic spines in response to glutamate via three main mechanisms of DAG lipase activation: one that is purely calcium-dependent, another that involves metabotropic Gq protein–coupled receptors, and a combined calcium assisted metabotropic mechanism [44]. In order to maintain correct levels of 2-AG concentrations, preventing unwanted inhibition of glutamate release and avoiding desensitization of CB1 receptor, this endocannabinoid is actively degraded by two hydrolases: post-synaptic ABHD6, and presynaptic MAG lipase (MAGL). In this frame, we suggested that in the hippocampus, peak 2-AG concentrations, required to counteract intense glutamatergic responses to social defeat stress are facilitated in mice by LSD1-mediated transcriptional repression of ABHD6 and MAGL [45].

LSD1, whose activity is transiently strengthened via stress-induced decreased levels of dominant negative isoform neuroLSD1, operates a negative modulation of ABHD6 and MAGL transcripts reflecting on protein availability within a homeostasis-demanding window of stress response [45]. Literature is concordant for what concerns 2-AG responses to acute stress, with many works reporting delayed (minutes to hours) 2-AG increase in the hippocampus, amygdala and prefrontal cortex as instrumental to neurotransmitter release regulation in these delayed windows of stress response [20,46,47]. Relevantly, 2-AG signaling in the hippocampus negatively regulates anxiety via positive modulation of DSE, but not inhibitory short-term plasticity [5], foreseeing a role for 2-AG increase as negative regulator of glutamatergic transmission. In general, hippocampal glutamatergic 2-AG signalling appears to be an essential component of adaptation to aversive situations [5].

LSD1-mediated repression of 2-AG degraders ABHD6 and MAGL probably cooperates with such an adaptation, contributing to increasing 2-AG tone in response to acute stress [45]. Indeed, stress-induced LSD1/neuroLSD1 ratio modulation in favor of LSD1 in the hippocampus occurs—requiring *de novo* transcription and functional modification of splicing factors—within hours. Although 2-AG has been shown to raise as early as half an hour after stress [20], LSD1 contribution to enhancing 2-AG levels via a posttranscriptional mechanism could be very relevant in situations of (i) prolonged stress, (ii) reiterated stress, (iii) during the late phases of stress allostasis, all consistent with the duration of 2-AG increase in the same area.

**Figure 1:**
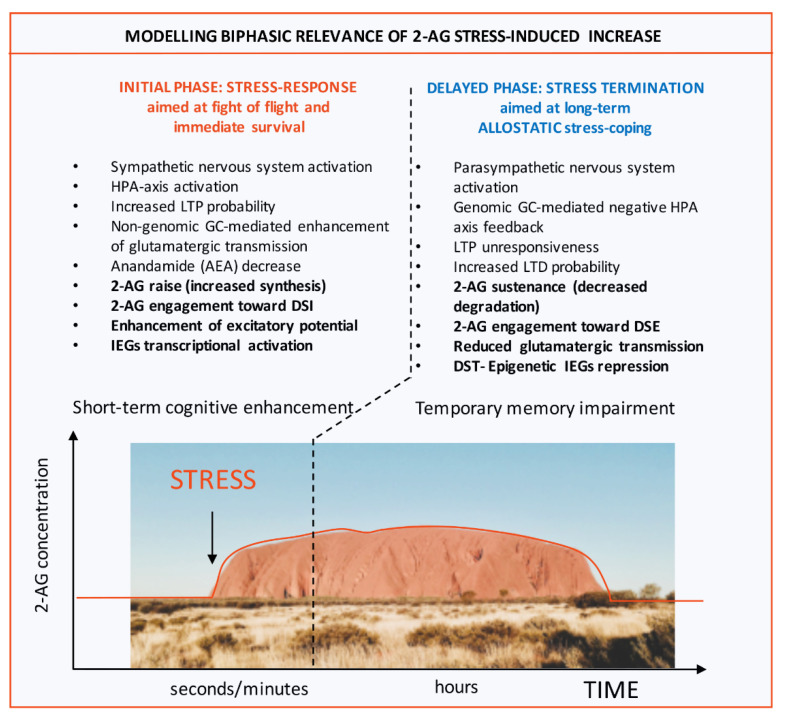
Modelling Biphasic relevance of 2-AG stress-induced increase and its epigenetic allostatic regulation as instrumental to stress response termination: an Ayer(s) perspective. The figure illustrates molecular and behavioral events occurring immediately upon stress perception (INITIAL PHASE) and in second phase in which primary responses, aimed at guaranteeing survival are gradually terminated (DELAYED PHASE). While in the first phase of stress response, which lasts seconds to minutes, cognition is boosted via neuroendocrine enhancement of glutamatergic transmission (short-term cognitive enhancement), in the second phase mechanisms underlying arousal as well as memory consolidation of the traumatic event are allostatically tuned down (resulting in a temporary memory impairment). Within this model, while in the first phase, endocannabinoid 2-AG increases its levels thanks to de novo synthesis by DAGLα, being involved in enhancing excitatory potential via Depolarization-Induced Suppression of Inhibition (DSI), in the second phase its tone is maintained elevated by an epigenetic process aimed at repressing transcription of the two 2-AG hydrolases MAGL and ABHD6. Thus, 2-AG displays a prototypic biphasic behavior. Notably, in this second window of stress response 2-AG is involved in decreasing glutamatergic transmission within the opposite form of endocannabinoid-operated short-term plasticity i.e. the process of Depolarization-Induced Suppression of Excitation (DSE). The graph displays enduring 2-AG stress-induced raise that lasts over both stress-response phases. The picture displays Ayers Rock (Uluru), Australia. Abbreviations used: 2-AG: 2-arachidonylglycerol; DAGLα: diacylglycerol lipase α; MAGL: monoacylglycerol lipase; ABHD6: alpha/beta hydrolase domain containing 6.

Interestingly, not only LSD1/neuroLSD1 ratio modulation and raise of 2-AG concentration are compatible with their cross-regulation, but these molecular responses to stress also take place within a window characterized by decreased ability of memory formation. This temporary interval of hippocampal inhibition that follows a very limited moment of cognitive enhancement after immediate stress perception, features LTP unresponsiveness in the frame of a widely-accepted protective role against excitotoxicity [27]. This stress-operated hippocampal depression was initially interpreted as an unwanted maladaptive effect of the traumatic event [48], while more recently, such refractory window of memory-consolidation has been endowed with adaptive significance as a behavioral stress-coping strategy. The first inherent hypothesis was that temporary impairment of memory formation could be instrumental to writing, via inhibition of all other potential memories, a perfectly-shaped long-lasting aversive memory of the traumatic event [49,50]. Another possibility we would like to push forward is that this effect could instead hamper memory consolidation of the traumatic event *itself*, favoring stress resiliency via limiting the formation of a too-vivid and detailed internal image of trauma, which relevantly also represents a *core* PTSD symptom. Indeed, even if in the *immediate early* stress-response window, 2-AG increase seems to be important to improve memory formation mediating DSI and hence enhancing memory retention of inhibitory avoidance training [51,52] (which is also consistent with the initial phase of cognitive enhancement [27]), in the delayed phase of stress response characterized by hippocampal inhibition—also featuring increased LTD probability likely with endocannabinoid contribution [53] — 2-AG could turn out to be necessary to DSE, contributing to temporary memory impairment or favoring extinction of the aversive memory instrumentally to resiliency [53]. For a graphical representation of biphasic 2-AG specificity see Figure 1. Thus, stress coping seems to display two opposing requirements the first being an immediate increase in cognition with protective purposes followed soon after, by the second requirement: to decreasing the quality of trauma-related memory traces (temporary memory impairment in Figure 1), again with the protective mean to limiting contextual anxiety arousal. Although biphasic 2-AG activity seems to be very likely it still requires a formal, comprehensive demonstration. A second relevant open question is whether LSD1-mediated MAGL and ABHD6 repression, functionally linked with increasing 2-AG [45] levels could be compatible with cognition enhancement or with a smemorizing effect. Considering the kinetic of LSD1-mediated MAGL and ABHD6 repression, which is delayed compared to initial stress perception, we suggest that LSD1 should be more functionally related with DSE promotion and more entrained with the window of hippocampal unresponsiveness. In this regard we can add that increased LSD1 activity is per se smemorizing [42] also displaying a prominent anxiolytic role in vivo [32].

## 5. Concerted Implications of ECS and LSD1 in Stress Vulnerability and Resiliency

Chronic homotypic stress triggers 2-AG increases in hippocampus, amygdala, mPFC, and hypothalamus [11]. Consistent results among different laboratories about 2-AG increase in the limbic system raise an important question related to the physiological relevance of this molecular response in the light of chronic stress-induced cellular and behavioral modifications. We anticipate that strong pieces of evidence suggest a protective role of 2-AG raise, prevalently played toward the glutamatergic system where this endocannabinoid stimulates CB1 receptor at excitatory axonal varicosities, leading to restraining glutamate release [6,54,55,56]. In this regard, 2-AG systemic supplementation not only decreases basal level of anxiety but also holds the remarkable behavioral implication to shifting the distribution of stress susceptibility towards resilience. Moreover, 2-AG further promotes resilience in previously susceptible mice. *Vice versa*, 2-AG depletion obtained by specifically inhibiting the activity of DAGL, converts stress resiliency into susceptibility [6]. Notably, this implies that 2-AG deficiency states could represent a stress susceptibility endophenotype and that 2-AG deficiency might contribute to stress-precipitated psychiatric conditions. Clinical relevance of 2-AG deficiency has been provided measuring circulating 2-AG content in the blood of a cohort of 9/11 terrorist attack, where those individuals meeting PTSD diagnostic criteria also showed significantly reduced 2-AG [57]. More specifically, it seems that the ventral hippocampus-basolateral amygdala (vHIP-BLA) glutamatergic circuit represents a nodal resiliency switch where 2-AG attenuation of glutamate release holds foremost beneficial outcomes [6]. All these findings have led to the suggestion that increased 2-AG CB1 receptor signalling may act as an endogenous stress-resilience factor that buffers against adverse consequences of stress [5,6,54].

These data are endowed with further significance thanks to the recent work of Nestler’s lab, who showed how LTD-like vHIP-Nucleus Accumbens (NAc) optogenetic modulation promotes resiliency to social defeat stress, while acute enhancement of this input induces stress susceptibility [58]. Moreover, another very recently described resiliency-instrumental strategy described by Hen’s lab, again implicates inhibition of excitatory dentate gyrus neurons of the vHIP [59]. In common, these results suggest that reducing vHIP E/I ratio is protective against negative behavioral drifts induced by chronic stress, notwithstanding efferent pathways are directed to BLA or NAc, or, more nodally, find themselves at an internal synaptic excitatory hippocampal system such as granule cells-CA3. It would be interesting to specifically assess whether 2-AG increase in the hippocampus in response to homotypic stressors, together with restraining vHIP-BLA circuit, also influences vHIP-NAc and granule cells-CA3.

The physiological relevance of 2-AG increase after multiple homotypic stress exposures could be enhanced by 2-AG accumulation due to increased, “on demand” synthesis, or reduced degradation. In this regard, our work suggests that negative transcriptional modulation of the 2-AG hydrolases MAGL and ABHD6 operated by LSD1 [45] could exert a cumulative effect along with reiterated stress sessions of the same kind [21], thus contributing to 2-AG augmentation by multiple waives of MAGL and ABHD6 transcriptional repression. The fact that inhibiting vHIP excitatory neuroplasticity promotes resiliency through efferent pathways directed to both the BLA and NAc, emphasizes the physiological relevance of stress-induced modulation of LSD1/neuroLSD1 ratio in favor of repressive LSD1 isoform in the mouse hippocampus [32,45]. Indeed, this molecular response, subserving negative regulation of excitatory neuroplasticity, sets upstream of multiple resiliency-like pathways. LSD1/neuroLSD1 ratio may therefore potentially act as a nodal system able to concomitantly regulating circuitry excitability instrumentally to resiliency [43]. LSD1, upon stress, downregulates two classes of gene targets: (i) activity-dependent-related targets involved in positive regulation of glutamatergic neuronal morphology and connectivity [42,43], and 2-AG degraders [45].

However, at a critical number of social defeat stressful sessions (ten or more), efficiency of neuroLSD1 splicing downregulation starts dropping, loosing transcriptional repressive strength over MAGL and ABHD6, which indeed are no more negatively modulated at the transcript level. These data suggest that desensitization of LSD1/neuroLSD1 protective mechanism might contribute to susceptibility-like hippocampal circuitry modifications. Allostatic overload has been suggested as an underlying cause of psychiatric drift. We suggest LSD1/neuroLSD1 splicing process as prototypic allostatic process suffering overload. Taken together, these data also suggest that decreased ability to reduce neuroLSD1 in response to stress [45] might contribute to 2-AG deficiency already observed in rodent models of chronic unpredictable stress (featuring neuropsychiatric stress-induced aberration after the 21st stress session) and 2-AG paucity in blood of PTSD-affected terrorism survivors [57].

## 6. Stress and tetrahydrocannabinol (THC) as Cannabinoid Signaling Desensitizers: Shared Epigenetic Language and Pathophysiological Implications

A significant molecular change that has been described after chronic stress exposure (i.e., chronic restraint stress or chronic unpredictable stress) is represented by reduction in CB1 receptor density, expression, and CB1-mediated synaptic signaling in several brain regions [12]. This maladaptive molecular response represents a sign of disrupted homeostasis at the basis of stress vulnerability. A similar cellular picture is also common to chronic cannabinoid exposure [60]. While in adult rats CB1 desensitization usually recovers within few days after the end of the treatment, when cannabinoid administration is performed in adolescent animals, changes involving the endocannabinoid system seem to be more intense and may last longer [61,62], thus exposing the young individual to longer periods of enhanced stress susceptibility. This evidence further highlights a relevant role for the ECS in neuroplastic mechanisms instrumental to stress coping. Accordingly, rats exposed to THC during adolescence develop depression-like behavior and different types of anxiety-like behavior when adult [63,64,65,66]. This behavioral picture is paralleled by alterations in brain neurocircuitry functionality, involving changes in gene expression [65,67]. The molecular underpinnings of these long-lasting effects may involve epigenetic mechanisms. Indeed, the epigenome contributes to providing a cellular context for environmental effects—including cannabis exposure—translating them into changes of gene expression [8]. Interestingly, we recently reported that adolescent THC exposure in female rats induces alterations in specific histone modifications in the PFC, which affected the expression of a set of genes associated with neuroplasticity, likely contributing to behaviorally-relevant circuitry maladaptation [65]. Complex kinetics characterize this process, with a first round of global increased trimethylation of H3K9, followed by a wave of histone H3 acetylation with an opposite outcome at the transcriptional level. H3K9me3 increases were likely mediated by the upregulation of Suv39H1, a histone methyltransferase specifically involved in the trimethylation of H3K9 [65]. A similar picture of histone modifications, with increased methylation of H3K9 and acetylation of H3K14 was also described in the hippocampus after the same adolescent THC exposure [68]. Remarkably, increased repressive histone mark H3K9me3 in the dentate gyrus was also shown after acute restraint stress, and this was obtained through an increase of the H3K9 methyltransferase Suv39H2 [69]. Similar epigenetic modifications observed after chronic THC exposure [65,68] seems to suggest that THC chronically administered to adolescent animals may recapitulate the same effects of chronic stress on limbic circuitry, thus impairing adaptive stress response. In other words, chronically administered THC increases vulnerability to develop stress-related psychiatric disorders.

## 7. Concluding Remarks

We first described glutamatergic nature of environmental stress stimuli at the level of corticolimbic emotional areas involved in stress processing, highlighting risks of hyperexcitation and circuitry disruption as substrates of psychopathologic aberrations. Then, we reviewed two homeostatic systems devoted to avoid glutamate load, the ECS and Epigenetic Homeostatic System (EHS). Interestingly, these two systems control each other, promoting stress adaptation and habituation. Finally, we posed on another interesting aspect of epigenetic-endocannabinoid interaction, emerging by the convergent effects of chronic stress and THC abuse on ECS disruption. Interestingly, a common epigenetic signature affects ECS functionality in case of stress- and drug-operated desensitization, switching stress response trajectory towards susceptibility.

The message we would like to deliver with this review article is related to the projective importance of a novel subfield of molecular psychiatry, which is focused on the clarification of synapse to nucleus crosstalk network involving epigenetic regulation of homeostatic synaptic systems and synaptic regulation of epigenetic mechanisms regulating availability of synaptic components [70].
